# Magnon-Magnon Interaction Induced by Dynamic Coupling in a Hybrid Magnonic Crystal

**DOI:** 10.1021/acsaelm.5c02128

**Published:** 2026-01-02

**Authors:** Rawnak Sultana, Mojtaba Taghipour Kaffash, Gianluca Gubbiotti, Yi Ji, M. Benjamin Jungfleisch, Federico Montoncello

**Affiliations:** † Department of Physics and Astronomy, 5972 University of Delaware, Newark, Delaware 19716, United States; ‡ CNR-Istituto Officina dei Materiali (IOM), Unità di Perugia, 06123 Perugia, Italy; § Dipartimento di Fisica e Scienze della Terra, 9299 Università di Ferrara, 44121 Ferrara, Italy

**Keywords:** patterned nanostructures, artificial spin ice, nanomagnonics, spin waves, magnonic crystal, magnon−magnon coupling, Brillouin light scattering spectroscopy, nanomagnetism, micromagnetism

## Abstract

We report a combined experimental and numerical investigation of spin-wave dynamics in a hybrid magnonic crystal consisting of a CoFeB artificial spin ice (ASI) of stadium-shaped nanoelements patterned atop a continuous NiFe film separated by a 5 nm Al_2_O_3_ spacer. Using Brillouin light scattering spectroscopy, we probe the frequency dependence of thermal spin waves as functions of applied magnetic field and wavevector, revealing the decisive role of interlayer dipolar coupling in the magnetization dynamics. Micromagnetic simulations complement the experiments, showing a strong interplay between ASI edge modes and backward volume modes in the NiFe film. The contrast in saturation magnetization between CoFeB and NiFe enhances this coupling, leading to a pronounced hybridization manifested as a triplet of peaks in the BLS spectra—predicted by simulations and observed experimentally. This magnon–magnon coupling persists over a wide magnetic field range, shaping both the spin-wave dispersion at fixed fields and the full frequency-field response throughout the magnetic hysteresis loop. Our findings establish how ASI geometry can selectively enhance specific spin-wave wavelengths in the underlying film, thereby boosting their amplitude and identifying them as preferential channels for spin wave transmission and manipulation.

## Graphical abstract

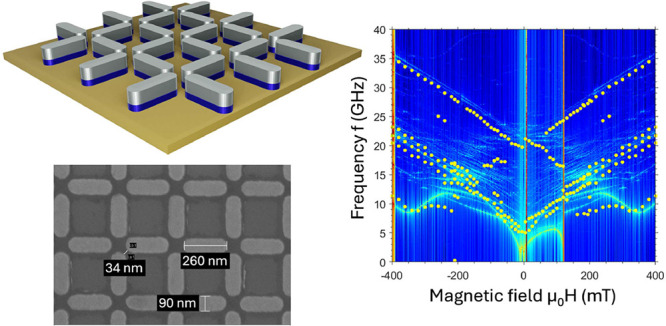

## Introduction

Artificial spin ice (ASI) structures consist of arrays of nanoscale magnetic elements arranged in geometrical patterns that mimic the frustration observed in natural spin ice.^1–3^ They represent a fascinating class of engineered metamaterials, specifically designed to control the spin-wave (SW) band structure, paving the way for the development of magnonic devices with reconfigurable magnetic properties, enabling tunable properties on demand.^4–9^ A critical aspect of ASI behavior is the strong magnon–magnon coupling effects,^10–12^ which arise due to strong dipolar coupling between adjacent nanomagnetic elements.^13,14^

In previous works, we studied the effect of NiFe ASI deposited on top of an unpatterned NiFe film with varying spacer layer thickness, composed of aluminum oxide (Al_2_O_3_),^14^ to understand how the dynamic stray magnetic fields generated by the ASI affect the SW propagation in an underlying NiFe thin film.^15,16^ In those works, ASI and film were made of the same material, hence having the same saturation magnetization (*M*_S_). This was found to facilitate the dynamic coupling between the film backward volume SW and several ASI bulk modes, leading to robust ASI–film hybrid modes that persist across variations in both field and thickness.

Here, we present a novel variation of the ASI/film hybrid structure employing different constituent materials to uncover new coupling phenomena. In the present work, the ASI is made of high *M*_s_ CoFeB, while the underlying thin film consists of soft NiFe, characterized by its comparatively lower *M*_s_. We reveal evidence for a magnon–magnon interaction between SWs in the film and the ASI edge modes, an interaction typically considered negligible due to weak dynamic stray fields associated with localized modes. The pronounced difference in the two material parameters displaces the ASI bulk mode frequencies relative to the film spin waves, effectively preventing their coupling. However, the ASI edge modes remain within the same frequency range as the film SWs, enabling distinct dynamic coupling. This coupling is not the result of a nonlinear excitation regime but is demonstrated, through simulations and experiments, to be intrinsically present in the system’s dynamics even for thermal magnons. In fact, the set of magnons of the CoFeB ASI layer and the spin wave of the continuous NiFe film do not evolve independently, as they are brought close together to form the bilayer but hybridize their profiles and shift their frequencies within a selected frequency range only. In this frequency range, the thermal spectrum of the hybrid structure is not merely the superposition of the ASI and film independent spectra, but a new distinct feature appears in the spectra in the form of a triplet, which we predict in the simulations and detected and characterized in the experiments. As a result, the otherwise negligible magnon–magnon interaction between edge modes and film SW is enhanced, thereby broadening the range of potential applications in reconfigurable magnonic devices and computing systems.

We use Brillouin light scattering (BLS) spectroscopy to measure the spectra of thermally excited SWs as a function of the magnetic field strength and wavevector. Furthermore, we employed micromagnetic simulations to interpret the experimental findings. This two-pronged approach allows us to reveal how the *M*_S_ difference between the ASI and the film can be effectively utilized to modulate the strength of the magnon–magnon interaction. In particular, since the saturation magnetization of a single layer can be tuned—for example, by laser irradiation^17–19^ —it becomes possible to engineer a magnonic device in which, through suitable material choice and design, the film mode can be dynamically coupled either to ASI edge modes or to bulk modes, depending on the irradiation.

Our work uncovers a new degree of freedom in designing tunable and energy-efficient magnonic systems, contributing to the emerging research field of 3D magnonics,^20^ where vertical stacking offers versatile coupling conditions for controlling SW propagation in patterned^21,22^ and unpatterned ferromagnetic films.^23–25^

## Materials and Methods

### Fabrication

The samples were fabricated by depositing a Ni_81_Fe_19_ (NiFe, Py) thin film onto a thermally oxidized silicon substrate by using electron beam (e-beam) evaporation. To introduce a nonmagnetic spacer layer, we deposit aluminum oxide (Al_2_O_3_) using the same e-beam evaporation technique. The ASI geometry is defined through electron beam lithography (EBL), followed by the deposition of Co_40_Fe_40_B_20_ (CoFeB) using e-beam evaporation. A subsequent lift-off process ensures precise patterning of the ASI structure. Further details on the fabrication procedure are provided in the Supporting Information of ref 14.

The fabricated samples are the following:

#1 A continuous NiFe film with a thickness of 20 nm.

#2 A continuous CoFeB film with a thickness of 20 nm.

#3 A square single-layer ASI lattice composed of 20 nm-thick CoFeB stadium-shaped nanoelements (islands).

#4 A hybrid structure consisting of a square ASI lattice (composed of 20 nm thick CoFeB islands) separated from a 20 nm thick NiFe film by a 5 nm thick Al_2_O_3_ nonmagnetic spacer layer.

The CoFeB ASI islands are designed as stadium-shaped elements with nominal lateral dimensions of 260 × 90 nm^2^. Figure 1a shows a graphical representation of the sample structure with emphasis on the vertical materials stack, while, in Figure 1b, a representative scanning electron microscopy (SEM) image of the ASI sample is shown. These islands are arranged in a square lattice, maintaining a minimum edge-to-edge spacing of 34 nm. The nominal lattice constant, defined as the center-to-center distance between neighboring nanomagnets belonging to the same sublattice, is *a* = 350 nm. This spacing determines the Brillouin zone boundary at a wavevector *k* = π/*a* ≈ 0.9 × 10^7^ rad/m.

**Figure 1:**
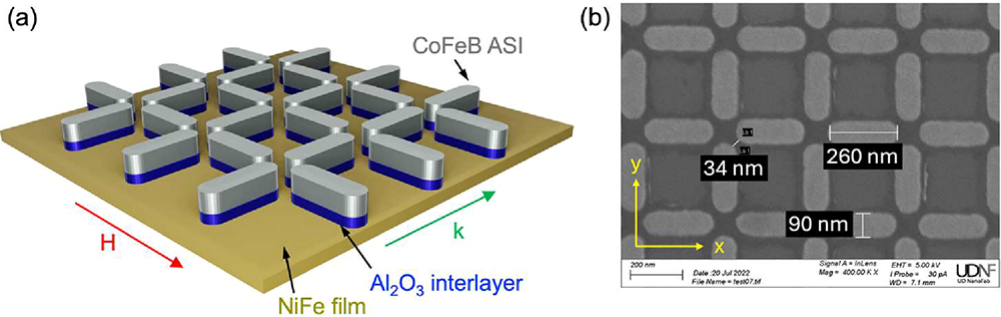
(a) Graphical representation of a sample structure consisting of CoFeB ASI deposited on top of a NiFe film separated by a 5 nm thick Al_2_O_3_ spacer. (b) Representative SEM image of the ASI sample along with the coordinate axes and the lateral dimensions of the ASI islands.

### Measurements

Magneto-optical Kerr effect (MOKE) magnetometry was used to measure the hysteresis loops of all samples in the longitudinal configuration, as discussed in the Supporting Information (SI).

Dynamic measurements were conducted using BLS. The BLS spectra were recorded at room temperature in a backscattering configuration.^26^ A monochromatic, p-polarized laser beam with a wavelength of λ = 532 nm and a power of approximately 200 mW was focused onto the sample surface at different incidence angles relative to the sample normal. The backscattered s-polarized light was then analyzed using a (3 + 3) tandem Fabry–Pérot interferometer,^27^ enabling high-resolution spectral measurements of the SW modes. A dc magnetic field was applied parallel to the sample plane and perpendicular to the incidence plane of light, which defines the direction of the SW wavevector *k*. The sample was mounted on a goniometer, enabling rotation around the field direction to vary the incidence angle of light (θ*_i_*) from 0° to 70°. Due to the conservation of in-plane momentum in the scattering process, the SW wavevector *k* is determined by the light incidence angle θ*_i_* and light wavevector $\frac{2\pi }{\lambda }$ , following the relation:

$$k=\frac{4\pi }{\lambda }\sin\theta_{i}$$ 1

This simple relation establishes a direct correlation between the experimental geometry (θ*_i_*) and the magnon wavevector (*k*), allowing for precise control over the probed SW entering the scattering process.

### Micromagnetic Simulations

Micromagnetic simulations of the magnetization dynamics for sample #3 and #4 were performed with the GPU-accelerated program Mumax3.^28^ The magnetic parameters were determined by fitting the experimental BLS curves of the Py and CoFeB films with the dipolar-exchange frequency-wavevector and frequency-field theoretical curves and are discussed in the SI. The micromagnetic elemental cell was set to 4 × 4 × 5 nm^3^, and each ASI island was represented by an oval shape with 64 × 20 cells, i.e., 256 × 80 nm^2^. For the simulations of the frequency/field curves and the mode profiles, a nonprimitive 2 × 2 unit cell was adopted to account for possible different magnetic charges (magnetization directions) at the ASI vertices,^33^ with an area of 704 × 704 nm^2^. We excited the system by a sinc pulse, with amplitude 1 mT and cutoff frequency 50 GHz. The simulation duration time was 20 ns, giving a frequency resolution of 0.05 GHz, while the sampling time step was 0.01 ns, giving the maximum (Nyquist) frequency of 50 GHz. The sinc excitation is applied to the relaxed, equilibrium magnetization at each applied field value, from −400 to +400 mT, in field steps of 2 mT. A slight tilt of 1° was applied between the applied field direction and one of the primary ASI axes to avoid any computational artifacts and account for unavoidable experimental error in field alignment.

For the frequency-wavevector dispersion at μ_0_ *H* = −350 mT, a primitive unit cell (352 × 352 nm^2^) could be used, since the large field allowed only two possible magnetizations, corresponding to the two orientations of the islands. In this case, to allow long wavelength phase variations, we used a supercell comprising 200 of the primitive cells, resulting in a wavevector resolution of ∼0.0089 × 10^7^ rad/m. The simulation time was 100 ns, corresponding to a frequency resolution of 0.01 GHz, while the sampling time step was 12.5 ps, corresponding to a maximum frequency of 40 GHz.

The simulated spectra show the square modulus of the out-of-plane component of the Fourier coefficients (in log scale), averaged between film and ASI layers in the hybrid system, while for the mode profiles (shown in Figures 4, 5, and 6) we plot the real part of the out-of-plane component of the Fourier coefficients (space-resolved phase amplitude),^35,29^ limited to the 2 × 2 nonprimitive unit cell.

## Results and Discussion

### General Features of BLS Spectra at Saturation

BLS spectra were acquired by sweeping the magnetic field μ_0_ *H* in the range from −400 to +400 mT at normal light incidence (determining a SW wavevector *k* = 0) and then by varying the wavevector *k* from 0 to 2.0 × 10^7^ rad/m at fixed μ_0_ *H* = −350 mT. Representative BLS spectra recorded at *k* = 0 (normal light incidence) and μ_0_ *H* = −350 mT for all the investigated samples are shown in Figure 2.

**Figure 2:**
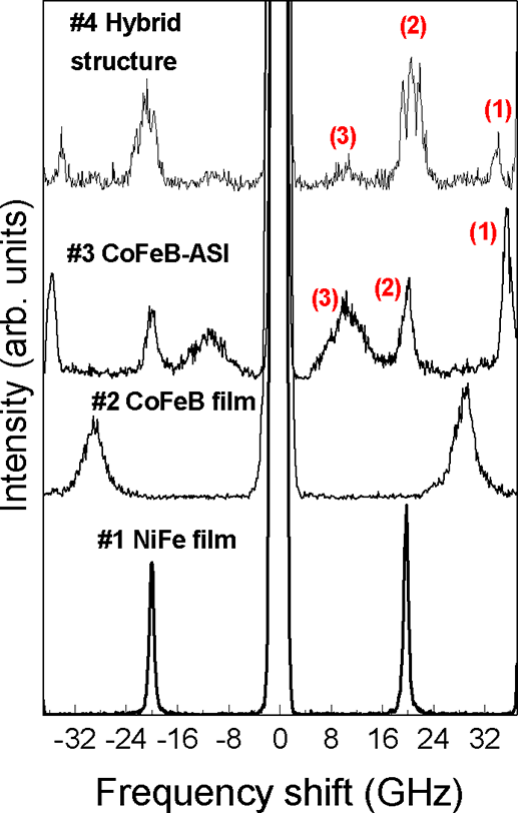
BLS spectra (intensity vs frequency shift) for all samples under investigation measured at normal light incidence (*k* = 0) and at a fixed magnetic field of μ_0_ *H* = −350 mT. Spectra are vertically shifted to highlight the different peak frequencies in the investigated samples. For samples #3 and #4, peaks are labeled by integer numbers 1, 2, and 3 to follow their field and *k* evolution and discussion in the text.

For the unpatterned NiFe and CoFeB thin films (samples #1 and #2, respectively), spectra exhibit a single prominent peak corresponding to the Kittel uniform mode (i.e., in the experimental BLS geometry, the Damon-Eshbach (DE) mode at *k* = 0). The frequency of these peaks is higher for CoFeB than for NiFe, primarily due to CoFeB’s larger saturation magnetization. Furthermore, the CoFeB film shows a significantly broader peak compared to NiFe, indicating increased damping or inhomogeneous line width broadening of the CoFeB sample.

In contrast, the CoFeB ASI structure (sample #3) exhibits three distinct peaks, labeled 1, 2, and 3. The two high-frequency peaks (1 and 2), located at approximately 35.5 and 20.2 GHz, are sharp and well-defined, whereas the low-frequency peak (3) is considerably broader, extending from about 7.5 to 13.5 GHz, indicating contributions from edge modes whose frequencies are sensitive to the island geometry, including shape, size, and edge roughness. Notably, peak (2) for the ASI sample (20.00 GHz) is very close in frequency to the peak observed in the NiFe film (19.65 GHz). This small frequency difference has important implications, which are presented and discussed in the following sections.

In hybrid sample #4, three peaks are also observed. The low-frequency peak at 10.2 GHz is notably broad and low in intensity. Upon closer examination, peak 2, located near 20 GHz, splits into a triplet of closely spaced peaks, indicating a more complex resonance structure. This splitting suggests the presence of additional mode interactions, likely resulting from the coupling between the localized modes of the CoFeB ASI islands and the propagating SWs in the NiFe continuous film, where the resonances of the film and ASI are nearly degenerate. This observation will be discussed in greater detail below.

### Frequency-Field Dependence of BLS Spectra

We begin by discussing the frequency-field dependence and comparing the experimental measurements to the simulated results (Figure 3). In the experimental BLS spectra, we tracked the field-dependent evolution of the magnonic peaks, shown at −350 mT in Figure 2, as a function of the applied field. For this purpose, we first saturated the sample magnetization by applying a strong external magnetic field of −400 mT along the *x*-direction. We then systematically ramped the field up to +400 mT in steps of 20 mT, following the ascending branch of the MOKE hysteresis loop shown in Figure S1 in the Supporting Information.

**Figure 3:**
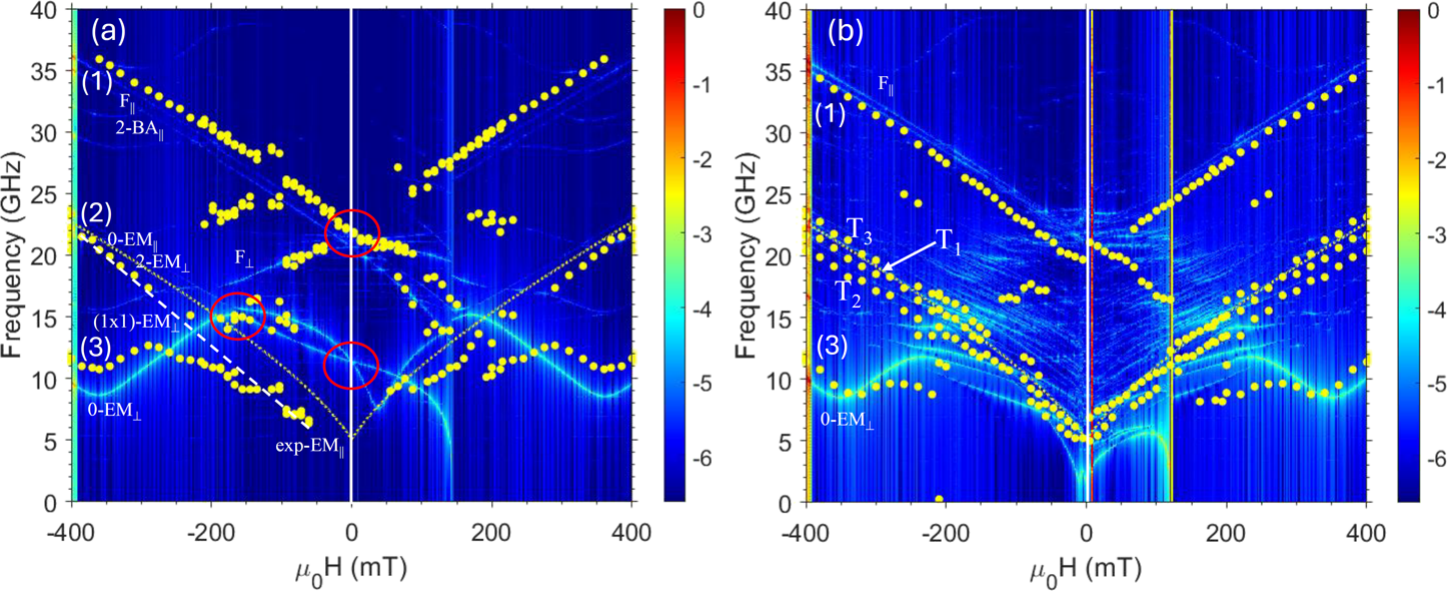
Field dependence (with increasing field from left to right) of the SW frequencies (full points) derived in the BLS spectra measured at *k* = 0 rad/m for (a) the CoFeB ASI sample (sample #3) and (b) the hybrid CoFeB ASI/Al_2_O_3_/NiFe film sample (sample #4). The color maps (in arbitrary units) represent the square of the average Fourier coefficients of all layers in the 2 × 2 supercell. The dotted line (symmetric for positive and negative field values) is the analytical curve of the Py film alone for comparison. Panel (a): the oblique dashed line serves as a visual guide, indicating the expected trend of the experimental data for 0-EM_||_; the leftmost red circle at −160 mT marks the first point at which the edge modes of both islands are degenerate in frequency in the simulation: this results in an increased intensity, which might correspond to the concentration of experimental data points observed in the same region. The rightmost red circles highlight the crossing points where edge (lowest circle) and fundamental (highest circle) modes become degenerate for all of the islands. Panel (b): hybrid modes resulting after ASI/film magnon–magnon interaction are labeled T_1_, T_2_, and T_3_, following the order in frequency and intensity found in the simulations, T_1_ being the most intense one. However, the experimental results, based on the measured BLS intensities, indicate that T_1_ and T_2_ must be interchanged.

### Frequency-Field Dependence of the ASI Sample

As a first step, we unveil the complex dynamics of sample 3 (single-layer ASI sample), being crucial to understand the dynamics of the ASI-film hybrid structure. At negative saturation, three peaks are observed in the BLS spectra, as shown in Figure 3a. Comparison with the simulations, based on the calculated mode frequencies and the spatial profiles of modes shown in Figure 4, suggests that these peaks correspond to the following modes (from highest to lowest frequency): the fundamental mode of the islands aligned parallel to the applied field [F_||_ in Figure 4f], the edge mode with zero nodes of the island parallel to the field [0-EM_||_ in Figure 4d], and the edge mode without nodes of the island perpendicular to the applied field [0-EM_⊥_ in Figure 4b]. The latter mode is particularly intense and extends into the central part of the island, likely due to hybridization with the fundamental mode of the same island. Interestingly, the fundamental mode of the island perpendicular to the applied field (F_⊥_) does not appear as a distinct, stand-alone mode at large negative fields. Instead, due to this hybridization, mode 0-EM_⊥_ effectively also serves as the F_⊥_ mode: this feature holds for a wide range of field values, until around −160 mT, when an independent F_⊥_ arises due to magnetization rotation (see below).

**Figure 4:**
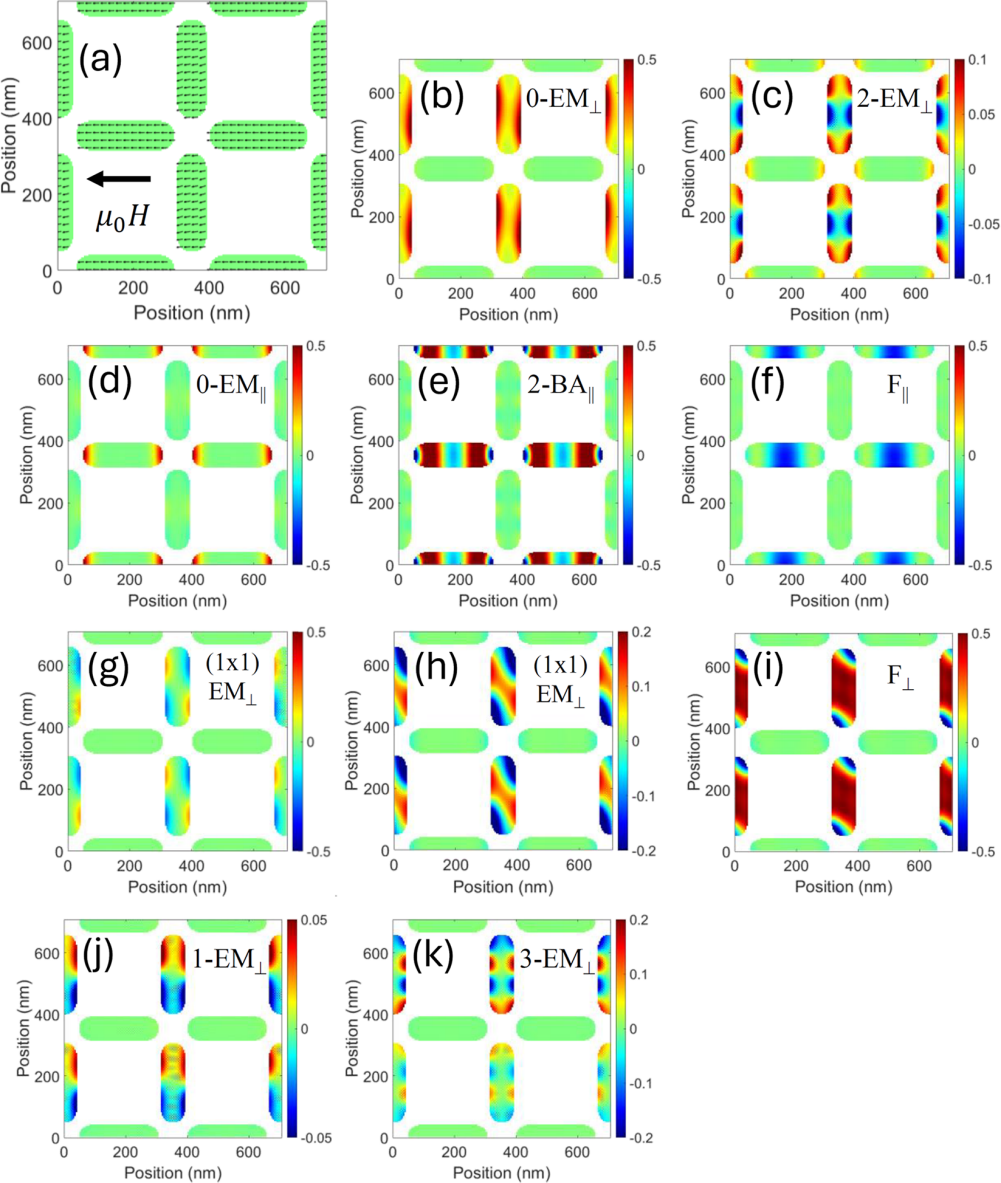
Magnetization ground state at μ_0_ *H* = −350 mT [panel (a), deliberately larger to make the arrows visible] and phase space profile of the main modes of the ASI system (b–k). Each profile corresponds to a 2 × 2 nonprimitive unit cell. Profiles (b–f) are calculated at μ_0_ *H* = −350 mT. Panel (b) represents mode 0-EM_⊥_, at 8.80 GHz, (c) is mode 2-EM_⊥_, at 20.90 GHz, (d) corresponds to mode 0-EM_||_, at 21.85 GHz, (e) is mode 2-BA_||_, at 33.45 GHz, and (f) is mode F_||_, at 34.40 GHz. Note that mode (c) is weakly hybridized with mode (d). In Panel (g–i), we show the evolution of edge mode (1 × 1)-EM_⊥_ to F_⊥_ as the underlying magnetization rotates: (g) 15.65 GHz, at −350 mT; (h) 17.3 GHz, at −250 mT; (i) 19.5 GHz, at −100 mT. Panels (j) and (k) show, correspondingly, the edge modes 1-EM_⊥_ (15.75 GHz) and 3-EM_⊥_ (25.5 GHz) calculated at −350 mT, which are usually of negligible intensity but become BLS active at large incidence angles (see text).

Since peaks (1) and (2) of sample #3 in Figure 2 originate from the islands parallel to the applied field, in which the magnetization distribution is almost unchanged up to −200 mT, they follow the typical linear Larmor frequency behavior ω = γμ_0_ *H* (in the simulations down to μ_0_ *H* = 0). Conversely, peak (3) stems from the island perpendicular to the applied field, whose magnetization undergoes a gradual reorientation (due to shape anisotropy), and hence it displays the typical “W-shape” behavior described in refs 30,31 : its curve intersects with peak (2) twice, at μ_0_ *H* = −160 mT and μ_0_ *H* = 0, as indicated by the red circle in Figure 3a.

In the simulations, a remarkable behavior is observed for the fully antisymmetric edge mode (1 × 1)-EM [i.e., an edge mode, with zero intensity in the bulk, having one node along either axis, Figure 4g]. Due to the gradual rotation of the underlying magnetization and the formation of an S-state configuration,^30,32^ this mode also undergoes a progressive transformation, acquiring a more symmetric profile. As the field approaches −160 mT (i.e., as a magnetization rotation of 45° is approached), the mode intensity in the bulk increases, and eventually, this edge mode transforms into a bulk mode, namely the new F_⊥_ (correspondingly, the 0-EM_⊥_ gradually loses intensity in the bulk, and hence the role of the fundamental mode). This effect appears to be confirmed by the experiments (Figure 3a), where a BLS signal emerges at around 19 GHz at −100 mT, in excellent agreement with the simulated frequency of the F_⊥_ mode. The evolution of this transformation is illustrated by the simulated mode profiles at −350, −250, and −100 mT in Figure 4g–i.

In the simulations, 0-EM_⊥_ and 0-EM_||_ intersect with one another at two field values: μ_0_ *H* = −160 mT (in Figure 3a, the leftmost red circle) and μ_0_ *H* = 0 (in Figure 3a, the lower red circle). At both fields, the magnetizations of the islands oriented parallel and perpendicular to the applied field are identical and, thus, indistinguishable. Specifically, at μ_0_ *H* = −160 mT, the magnetization in all islands (regardless of their orientation) adopts an S-state configuration with a 45° angle relative to the applied field, as previously discussed (the slight apparent anticrossing is due to the slight inequivalences of the two S-states due to the serrated shape of the islands, an artificial effect due to the micromagnetic discretization). At μ_0_ *H* = 0, the magnetization aligns along the long axis of each island, again making the two orientations equivalent. Similarly, F_⊥_ and F_||_ become degenerate at μ_0_ *H* = 0 (in Figure 3a, the higher red circle), where the magnetization aligns along either island long axis, hence making them indistinguishable. Considering these degeneracy effects, the latter, involving the fundamental modes, is clearly detected in the experiment (Figure 3a, the higher red circle) at a frequency of about 20.5 GHz. In contrast, the former degeneracy, involving the edge modes, is less evident in the experiment [lower red circle in Figure 3a]. In fact, if we inspect the BLS measurements only (symbols in Figure 3), the first edge-mode intersection appears to occur at a lower field, i.e., around μ_0_ *H* = −250 mT (−160 mT in simulations), where the extrapolated trend of the 0-EM_⊥_ points seems to intersect that of 0-EM_||_ (Figure 3a), dashed oblique line). If this were true, the experimental data would fail to reproduce the second EM degeneracy, expected at μ_0_ *H* = 0: the trend of the dashed oblique line (0-EM_||_) would intersect the frequency axis (μ_0_ *H* = 0) at approximately 3.5 GHz, appearing disconnected from the trend inferred from the experimental points measured within the interval [−150, −100] mT at around 15 GHz for the 0-EM_⊥_ mode.

Despite being difficult to unambiguously interpret, we justify this discrepancy by recalling that edge modes are typically very sensitive to the geometrical parameters (shape, edge roughness, spacing, etc.): therefore, because of minor mismatches between simulated and real samples, the measured frequency/field slope of mode 0-EM_||_ appears to significantly depart from the simulated trend, and the actual BLS intensity of these modes appears below the noise level.

In the simulations, we also observe higher-order modes, which are close in frequency to those of F_||_. These modes exhibit *m* nodes perpendicularly to the magnetization and are identified as “backward” modes (*m*-BA) akin to the backward-volume SW configuration.^16,33^ The most intense among these are the modes with the lowest even values of *m* [*m* = 2 in Figure 4e]. In the real sample, these higher-order modes may merge, resulting in a broad peak (1) observed in the experimental spectra in Figures 3 and 4.

Finally, we observe that mode 2-EM_⊥_ (Figure 4c) is close in frequency (less than 1 GHz) and weakly hybridized with mode 0-EM_||_ (Figure 4d). This proximity will be significant in the following discussion, where we examine the coupling between the ASI and film modes. Here, we want to recall how ASI modes generally arise as linear combinations (superpositions) of the normal modes of the individual (isolated) nanoelements (islands), which serve as the fundamental building blocks for the interacting ASI system.^31^ The dipolar interaction among the ASI islands introduces a perturbation that couples the single-island wave functions and shifts their frequencies.

### Frequency-Field Dependence of the ASI-Film Hybrid Structure

The overall frequency-field dependence of the ASI-film hybrid structure (sample #4), as shown in Figure 3b, closely resembles that of the isolated ASI structure, particularly in the frequency ranges relevant to peaks 1 and 3. We argue that these peaks are due to excitations confined to the ASI layer only, a point we substantiate further below. However, an intriguing feature emerges for peak 2, where a triplet of peaks, labeled as T_1_, T_2_, and T_3_, is observed over a large field range instead of the single peak measured for the ASI structure. The label order, T_1_, T_2_, and T_3_, follows the order in frequency and intensity found in the simulations, with T_1_ being the most intense one (also dispersive, as will be presented below). However, the experimental spectra of Figure 7b indicate, based on the measured BLS intensities, that T_1_ and T_2_ must be interchanged. This explains why T_1_ appears in the middle of the triplet in Figure 3b, as it will also in Figure 8b.

The experiments evidence that the (thermal) BLS spectra of the hybrid ASI-film structure are not just the superposition of the spectra of the independent ASI and film layers. This observation manifests the intrinsic nature of the interlayer magnon–magnon coupling facilitated by dynamic dipolar coupling^10^ and not by any power-dependent nonlinear excitations of the magnon system. In a specific frequency range, the interaction leads to a hybridization of ASI and film mode profiles, creating interlayer hybrids that result in frequency shifts of the unperturbed modes in the isolated systems.

The frequencies of peaks T_1_, T_2_, and T_3_ evolve linearly with the applied field, and their frequency separation remains nearly constant within the detectable frequency range: in the experimental BLS measurements, approximately 1.2 GHz between T_1_ and T_2_ and 1.5 GHz between T_1_ and T_3_. This observation, along with the fact that the measured spectrum of the hybrid structure is not merely a superposition of the spectra from the CoFeB ASI and the NiFe film, is an experimental consequence of both the static and dynamic coupling between the ASI and film layers. Regarding static coupling, the ASI nanoelements retain nearly the same magnetization as in the isolated system, owing to their strong shape anisotropy. In contrast, the film layer is significantly influenced by the presence of the ASI layer (Figure 5d, bottom panel). Not only does the film acquire the periodicity of the ASI lattice, but it also develops a remarkable magnetization inhomogeneity at the edges of the primitive cell, i.e., in correspondence to the ASI vertices. This is due to the dipolar interaction, which leads to an overall reduction of all magnon frequencies.

**Figure 5:**
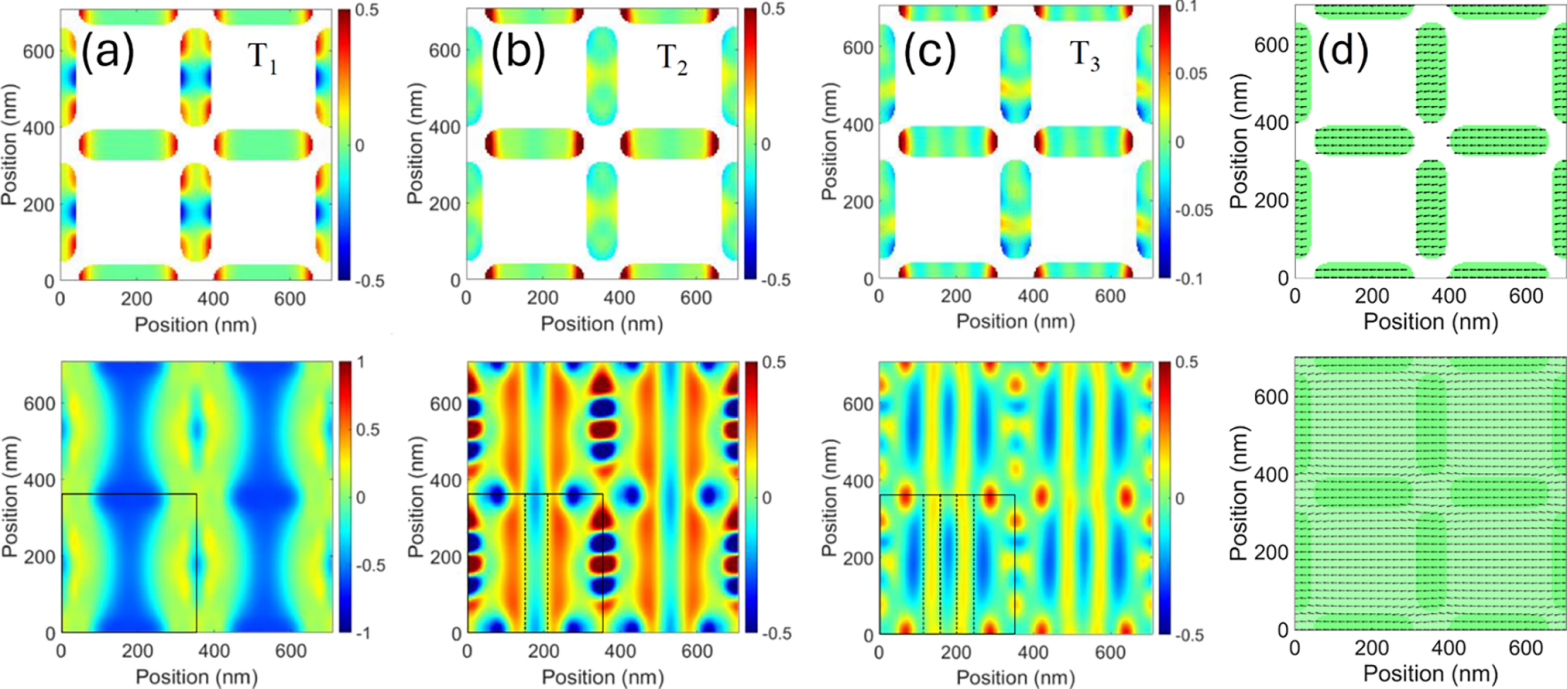
Simulated phase space profile of the triplet T_1_ (a), T_2_ (b), and T_3_ (c), emerging in the hybrid structure at μ_0_ *H* = −350 mT (colormap is in arbitrary units). Each profile corresponds to a 2 × 2 nonprimitive unit cell. Top panel: CoFeB ASI layer modes; bottom panel: NiFe film layer modes. Note that the excitation phase profile in the ASI layer is almost the same for all modes T_1_, T_2_, or T_3_, while the film layer phase profiles show an increasing number *m* of nodes (*m* = 0, 2, 4) in the *x*-direction, selecting *k* = 0, *k* = 3.57 × 10^7^ rad/m, *k* = 7.14 × 10^7^ rad/m (with *k* parallel to μ_0_ *H*), respectively, with frequency (a) 20.25 GHz (T_1_); (b) 21.45 GHz, (T_2_); (c) 23.55 GHz (T_3_). The square box on the left-bottom of each panel marks the primitive unit cell, and the dotted lines illustrate the nodal lines. Panel (d) shows the static magnetization distribution of the two layers at μ_0_ *H* = −350 mT. The film layer shows the shadow of the ASI islands as a guide for the eye.

In terms of dynamic coupling, simulations of our hybrid system show that it occurs exclusively between the ASI island edge modes and the film backward-volume mode, confined to a narrow frequency window around 22 GHz. More specifically, the two ASI modes 2-EM_⊥_ and 0-EM_||_ [in the isolated ASI, at 20.9 and 21.85 GHz, Figure 4c,d] “merge” (i.e., they are excited at the same frequency) in the ASI layer of the hybrid structure and couple with the NiFe film mode (occurring for *k* = 0 at 20.25 GHz in the isolated thin film), resulting in a triplet at (a) 20.25 GHz, (b) 21.45 GHz, (c) 23.55 GHz (Figure 5). As can be seen from Figure 5, the phase profiles of the ASI layer for these three modes are rather similar (i.e., a superposition of modes 2-EM_⊥_ and 0-EM_||_), while the film layer of the same structure exhibits an increasing number of nodal lines (i.e., wavevector) along the direction of the applied field: the nodal lines precisely correspond to the phase distribution observed in the ASI layer. This observation manifests a unique type of magnon–magnon coupling, which can be interpreted as a form of hybridization that persists over a wide range of applied magnetic fields (from −400 mT to −100 mT in our system). This magnon–magnon coupling is enabled by the distinct edge mode characteristics of the horizontal and vertical sublattice sites (islands), their interaction in the presence of the underlayer film, and their coupling with the mode of the NiFe underlayer. Interestingly, this process leads to an excitation of higher wavevectors, which would not be possible to excite without the ASI in the system.

The hybrid mode T_1_ at 20.25 GHz [Figure 5, panel a] has no full nodal lines in the film layer (bottom panel), while T_2_ at 21.45 GHz [panel (b)] has (with reference to the primitive unit cell) two nodal lines. Finally, T_3_ at 23.55 GHz has 4 nodal lines. The number of nodes in the film layer, which in this case is along the direction of the applied field, determines an effective wavelength of infinite, a half and a quarter of the unit cell, respectively, from which we obtain the effective wavevectors *k* = 0,^34^ *k* = 3.57 × 10^7^ rad/m, and *k* = 7.14 × 10^7^ rad/m. By inserting these wavevector values in the dipole-exchange analytical dispersion of the backward volume SWs (i.e., with wavevector parallel to the applied field),^35^ we obtain the expected frequencies for the corresponding uncoupled (uniform) film, namely, 20.32, 19.96, and 22.55 GHz, respectively. This analysis enables us to determine the frequency region where the coupling occurs as well as the frequency spread of the coupled modes. The magnon–magnon interaction can thus be interpreted as a perturbation that mixes the original mode profiles and shifts their frequencies: this concurrent effect, which is dependent on the frequency range of interest, is a form of magnon–magnon interaction not originating from high-power nonlinear SW excitations.

### Dynamic Coupling Mechanism

Now we pause the discussion to outline the general theory of the dynamic coupling.^14,16^ In general, the excitations of two coupled magnetic layers remain independent when external magnetic or geometric parameters, such as the applied field or film thickness, are varied. However, under specific conditions,^14,16^ a dynamic coupling emerges between the excitations of the two layers, manifesting as an interaction among two or more magnons that remains robust against changes in geometry or magnetic field variations. In such cases, a specific ASI layer mode profile couples to a specific film layer mode profile at the same frequency so that both are excited simultaneously—in other words, they become “locked.” This coupling persists over a specific parameter range that scales with the interaction strength. Consequently, the extent of this range provides an indirect experimental measure of the coupling. The resulting magnon–magnon interaction leads to hybridization of the ASI and film modes, potentially giving rise to distinct acoustic and optical branches. As discussed in more detail in refs 14,16 , the dynamic coupling is facilitated by mode hybridization and is most likely to occur between mode profiles with compatible symmetry, i.e., the same distribution of nodal lines (same odd/even number of nodal lines) or relative phase. Notably, proximity in the frequency of modes is a necessary but not sufficient condition for dynamic coupling to take place.

### ASI-Film Hybrid Modes Not Dynamically Coupled

The additional experimentally observed BLS peaks, specifically peaks (1) and (3) in Figure 3b, have so far been left out of the discussion. The comparison with the simulations, based on proximity in frequency and similarity in the slopes of the curves, provides insights into their origin. The lowest frequency peak (3) is attributed to mode 0-EM_⊥_ [Figure 6a]. Despite being classified as an edge mode, it exhibits considerable intensity. As evident from the calculated profile, the amplitude is strongly enhanced not only at the field-aligned edges—where the effective field exhibits deep minima—but also at the island center, indicating hybridization with the fundamental mode. Its field behavior [Figure 3b] closely resembles that observed in the ASI alone, suggesting that it still originates from the ASI layer only (whose magnetization distribution is barely affected by the static coupling with the film). The associated film layer does not contribute to the overall excitation. In fact, by inspection of the corresponding film layer [Figure 6a bottom panel], we recognize only negligible dynamic magnetization. The only effect of the film layer on the ASI is a slight frequency downshift, from 8.8 to 8.6 GHz, due to the *static* dipolar interaction between the layers.

**Figure 6:**
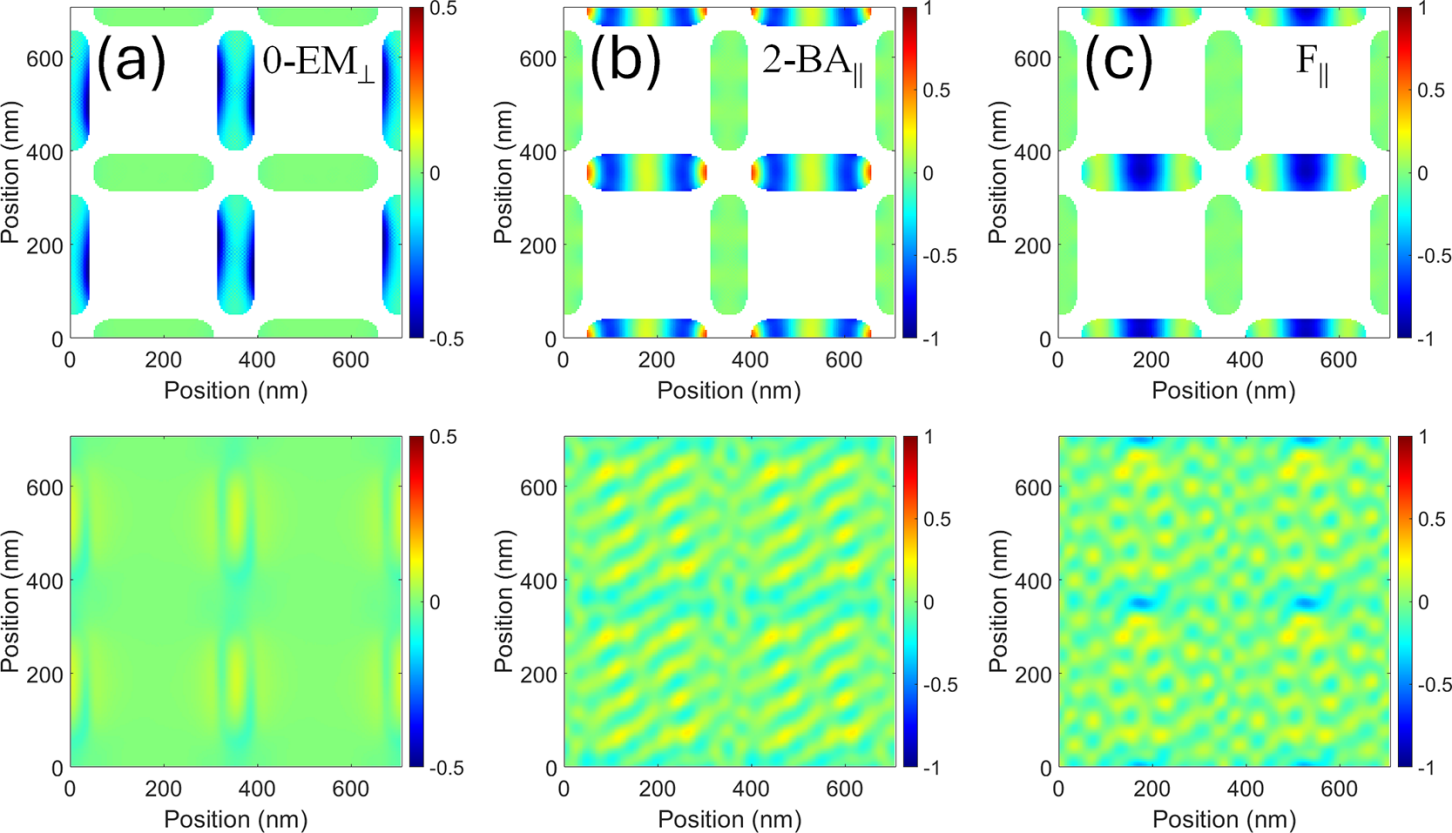
Phase profile of the top and bottom layers in the hybrid ASI/film structure calculated at an applied magnetic field of −350 mT. Top layers show the phase profiles of ASI modes (a) 0-EM_⊥_, at 8.60 GHz; (b) 2-BA_||_, at 33.6 GHz; and (c) F_||_, at 34.2 GHz. In all three cases, the bottom layer remains unresponsive, although subjected to microwave excitation, it exhibits negligible dynamic magnetization, suggesting that ASI and film are dynamically uncoupled at these frequencies.

Similarly, peak (1), which appears at the highest measured frequency in Figure 3b, exhibits nearly the same absolute values and frequency-field dependence as in the uncoupled ASI (Figure 3a), indicating that it originates solely from the ASI layer. By comparison with the simulations, we attribute peak (1) to the modes shown in Figure 6b,c, which lie close in frequency and can therefore explain the observed peak broadening. These correspond to bulk modes 2-BA_||_ and F_||_ in the ASI layer. In contrast, the film layer remains essentially unexcited, as evidenced by the negligible dynamic magnetization in its profile. The only contribution of the film is a slight frequency shift due to static (dipolar) coupling: mode (b) is upshifted from 33.45 to 33.6 GHz, while mode (c)—the ASI fundamental—is downshifted from 34.4 to 34.2 GHz.

### Frequency-Wavevector Dispersion Curves for the ASI System

In Figure 7a, a sequence of BLS spectra taken at different *k* is shown. In the CoFeB ASI system, three prominent peaks, labeled as 1, 2, and 3, are observed in the low-wavevector range. As already discussed above, these peaks correspond to ASI mode F_||_, EM_||,_ and EM_⊥_, respectively. Additionally, two more peaks (4 and 5)—located at 14.7 and 24.36 GHz—appear when the wavevector *k* exceeds 0.73 × 10^7^ rad/m. At *k* = 0, the lowest-frequency peak, centered around 10.2 GHz, is relatively broad, whereas the peaks at approximately 20 and 35.5 GHz are sharper and more well-defined. To explain the appearance of additional peaks at higher wavevectors, it is important to consider the wavevector dependence of the BLS signal, which is governed by the spatial distribution of the dynamic magnetization. BLS measurements can be interpreted within the framework of the so-called *Fourier microscopy approach*: at small wavevectors (i.e., θ*_i_* close to zero), the BLS scattering cross-section is proportional to the squared modulus of the Fourier transform of the out-of-plane component of the dynamic magnetization.^36–39^

**Figure 7:**
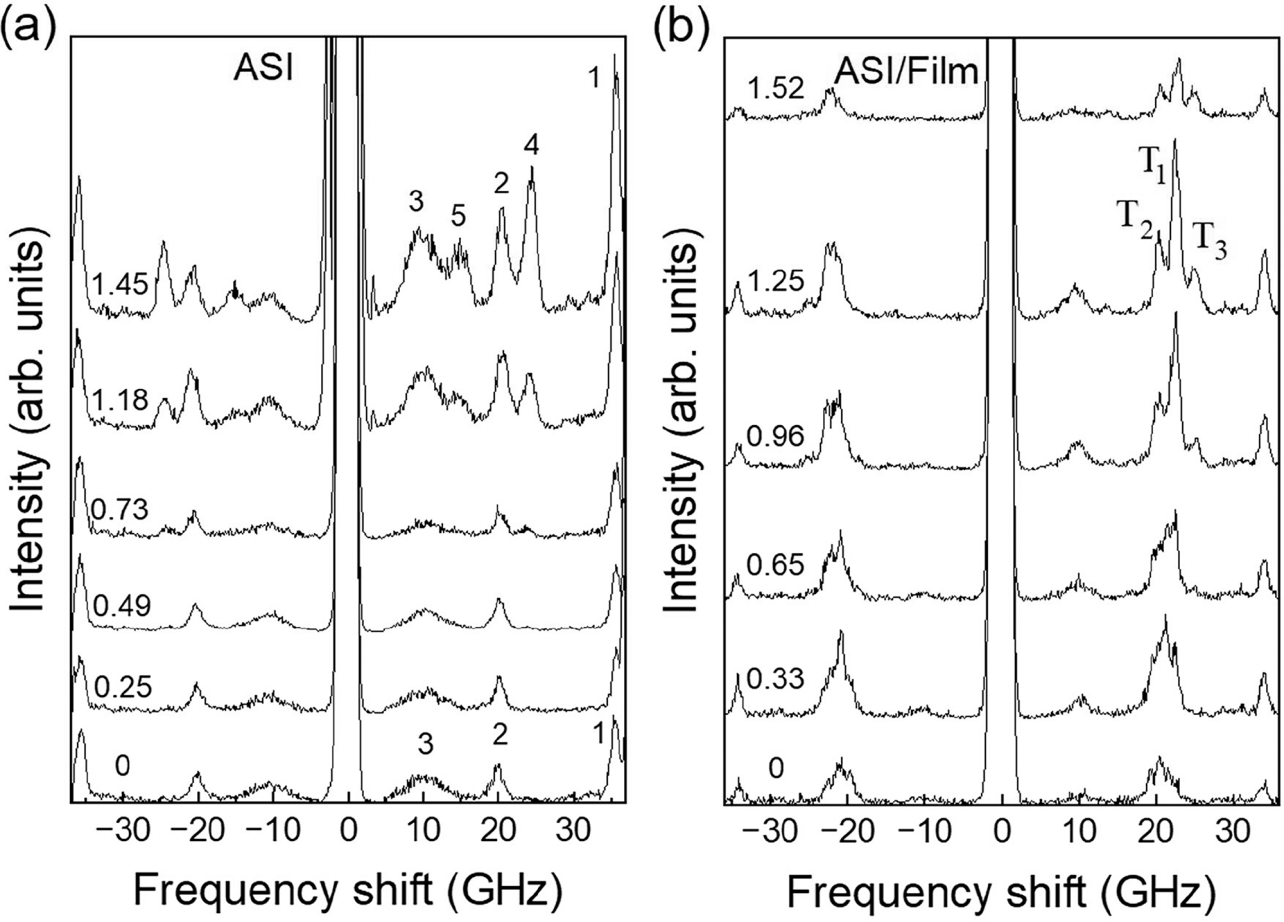
Comparison between wavevector-resolved BLS spectra of (a) single-layer CoFeB ASI (sample #3) and (b) CoFeB ASI/NiFe film hybrid structure (sample #4) at μ_0_H = −350 mT applied along the ASI symmetry direction. The numeric labels of each spectrum (on the left side) indicate the corresponding *k-*values (in units of 1 × 10^7^ rad/m). Numeric and alphabetic labels on the right side correspond to the peaks discussed in the text.

Propagating modes in a periodic medium can be expressed as Bloch waves because of the translational symmetry:^40,41^

$$\partial m_{k}(r)=\partial {\tilde{m}}_{k}(r)e^{ikr}$$ 2

where ∂*m*_*k*_ is the actual dynamic magnetization, ∂*m̃* *_k_* (*r*) is the cell function (limited to the primitive unit cell), *r* is the position across the lattice, and *k* is the SW wavevector (in our case, perpendicular to the applied field). The BLS mechanism detects the out-of-plane component of ∂*m*_*k*_(*r*), which varies with the wavevector according to eq 2. It was shown in ref 38. that the BLS amplitude can be written as a product of a form factor (calculated over the illuminated area and hence involving many primitive unit cells and depending on the primitive lattice vector ***R***) and a structure factor, calculated at ***R*** = 0 limited to a single primitive unit cell. For a sufficiently large illuminated area, the form factor reduces to δ­(Δ*q* – *k*) where

$$\Delta q=\frac{4\pi }{\lambda }\sin\theta_{i}$$

is the in-plane transferred wavevector of the probing light beam, and *k* is the magnon wavevector, and δ is the Dirac delta function. This implies that *k* = Δ*q*, which corresponds to the matching of the in-plane transferred photon momentum and the magnon momentum. The structure factor, on the other hand, is limited to a single primitive unit cell (***R*** = 0), where ∂*m*_*k*_(*r*) ≡ ∂*m̃* *_k_* (*r*), and reads:

$$S=\int_{cell}\partial {\tilde{m}}_{k}(r)e^{i\Delta q\cdot r}dr$$ 3

The final BLS intensity is *I*_BLS_ ∼ |*S*|^2^ if *k* = Δ*q*.

For modes with an even phase profile, ∂*m̃* *_k_* (*r*), the BLS signal is the strongest near the normal incidence (i.e., around θ*_i_* = 0°), and such modes are visible over a wide range of incident angles. In contrast, modes that exhibit an odd phase profile in the wavevector direction, characterized by an effective wavelength Λ and wavevector $k=\frac{2\pi }{\Lambda }$ , lead to a resonant enhancement of the BLS signal at specific incidence angles. As such, the emergence of high-*k* peaks in the spectrum can be attributed to any modes that become optically accessible only at larger incidence angles, corresponding to larger transferred wavevectors.

The mode profiles we show in Figures 5, 6, and 7 correspond to a 2 × 2 repetition along *x* and *y* of the real part of the *z*-component of the SW mode cell function ∂*m̃* *_k_* (*r*), i.e., the SW mode profile ∂*m*_*k*_(*r*) at *k* = 0. When *k* (i.e., θ*_i_*) is increased, the exponential Bloch factor in eq 2 introduces a progressive phase shift in ∂*m*_*k*_(*r*), which gradually changes the symmetry of the SW profile ∂*m*_*k*_(*r*), and hence impacts the BLS cross section and overall signal intensity as discussed above.^38,39^

Comparison with the simulations helps to associate peaks 4 and 5 (measured at 14.7 and 24.36 GHz at −350 mT) with edge modes having an odd number of nodes perpendicular to the applied field, specifically mode 1-EM_⊥_ and 3-EM_⊥_ [Figure 4j,k, calculated at −350 mT at 17.75 and 25.5 GHz, respectively]. These modes are localized in islands oriented perpendicular to the field, where oscillations exist along the island long axis. If these modes were instead confined to islands parallel to the field, the oscillations would have been confined within the island short axis, resulting in significantly higher frequencies. The ASI modes 1-EM_⊥_ and 3-EM_⊥_ exhibit odd cell functions ∂*m̃* *_k_* (*r*) because those modes have odd nodal lines perpendicular to the field. For an effective SW wavelength Λ that equals the lattice constant of the artificial spin-ice lattice *a* = 352 nm, the Bloch wavevector is $k=\frac{2\pi }{a}$ : for those odd modes, this results in an even Bloch SW profile ∂*m*_*k*_(*r*), because at $r=\frac{a}{2}$ , the product of the phase factor *e* *^ikr^* and ∂*m̃* *_k_* (*r*) is positive [see eq 3], which means the probing light for a particular incidence angle and the magnon are in phase, leading to a resonant enhancement of the signal. However, in the experiment, the BLS cross section appears to become nonvanishing well below this value: in the experiments [Figure 8a], this enhancement begins already across the first Brillouin zone boundary.

**Figure 8:**
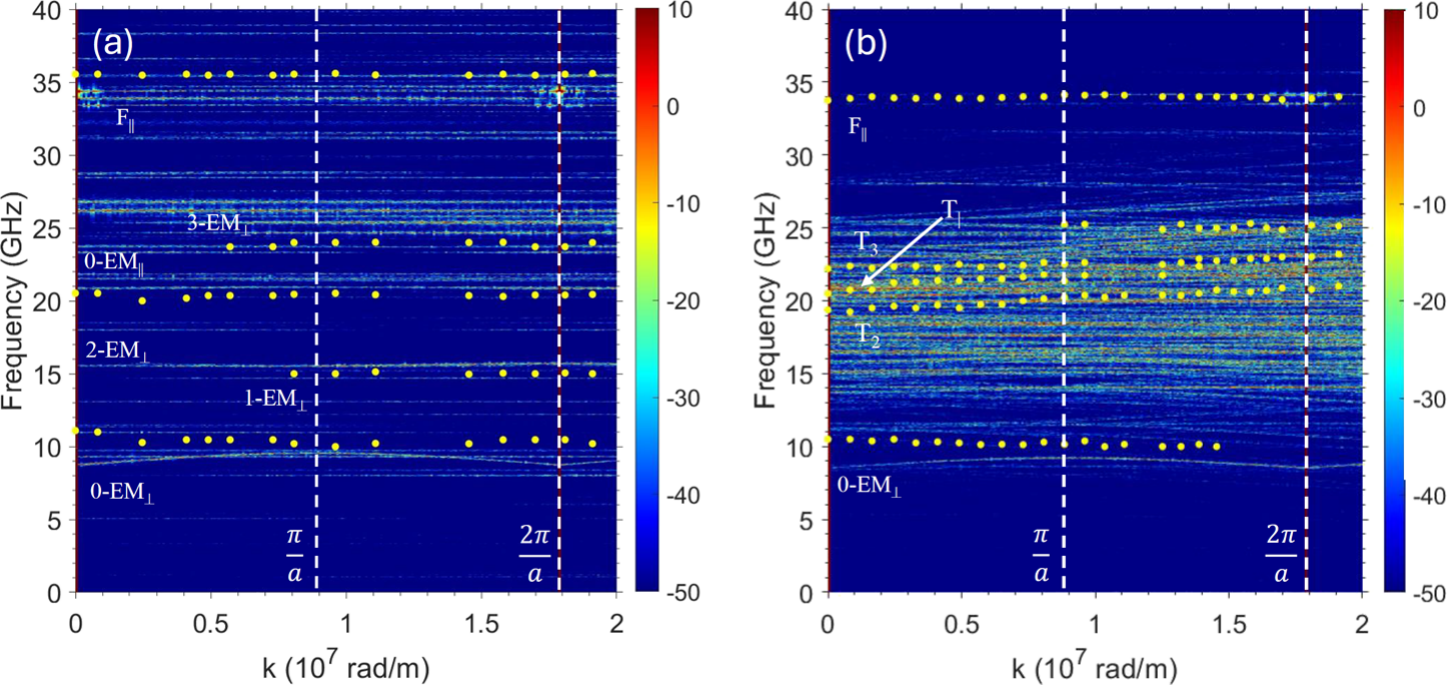
Comparison between the measured (data points) and simulated (color map) SW frequency dispersion for the ASI [panel (a)] and ASI-film hybrid [panel (b)], for an applied field μ_0_ *H* = −350 mT. The color bar on the right side of the panels is the intensity of the Fourier coefficients in logarithmic arbitrary units. The vertical dashed lines mark the boundaries of the first and second Brillouin zones. A propagating mode (i.e., with nonvanishing group velocity) appears in the hybrid system with frequency around 20 GHz at *k* = 0 (indicated by the arrow), called T_1_ in the text.

Figure 8 compares the measured and simulated dispersion relation between 0 and 2.0 × 10^7^ rad/m corresponding to the first and second Brillouin zones in the reciprocal space for the ASI system (sample #3) and the hybrid sample (sample #4). For the ASI sample, all modes are dispersionless, as is shown in panel (a); that is, their frequencies remain constant with varying wavevector *k*, indicating that the SW modes do not propagate through the ASI structure due to the rather small interelement dynamic coupling between the modes originating from the vertical and horizontal islands.

### Frequency-Wavevector Dispersion Curves for the Hybrid ASI-Film Structure

Inspection of the BLS sequence of spectra in Figure 7b indicates that the lowest frequency mode (9.7 GHz) and the highest frequency mode (34.0 GHz) exhibit minimal variations across the measured *k* range and suggest they originate from stationary modes of the ASI layer (in agreement with the other evidence discussed above). The peak at 9.7 GHz is broad and relatively weak in intensity: both features are consistent with the “edge” character of mode 0-EM_⊥_ in Figure 6a found in the simulations. The peak at 34.0 GHz is sharp and well-defined, consistent with the “bulk” character of mode F_||_ shown in Figure 6c.

Conversely, the mode triplet observed between about 18.7 and 22.9 GHz exhibits a significant frequency evolution as *k* increases, with the frequency separation between peaks increasing with *k*. This trend is particularly noticeable on the anti-Stokes side of the spectra, as can be inferred from the spectra presented in Figure 7.

Comparison with simulations helps to associate the mode with the largest dispersion slope to the T_1_ hybrid mode shown in Figure 5a, while the other two, less dispersive modes are identified as T_2_ and T_3_ modes [Figure 5b,c], respectively. This interpretation is supported by the fact that a higher number of nodes in the film layer leads to a reduced dynamic stray field and, consequently, narrower bandwidths. The same argument justifies the largest intensity of the T_1_ BLS peak, mentioned previously. In the simulations, the two modes T_2_ and T_3_ appear at higher frequencies than T_1_, while in the experimental data, based on their dispersion slopes, they appear above and below the frequency of the T_1_ mode.

## Conclusions

We report the hybridization of a backward SW mode in a ferromagnetic film with edge modes of an ASI system resulting from the dynamic coupling between layers made of different magnetic materials. Such hybridization manifests as a mode triplet in the BLS spectra. We interpret this triplet as a distinctive magnon–magnon interaction, facilitated by the large magnetic contrast between NiFe and CoFeB, i.e., the difference in the *M*_*s*_ values. As revealed by an extensive analysis of the frequency-field dependencies, this interaction is robust over a wide range of applied magnetic fields. In the dispersion relationships, the triplet of modes exhibits a propagating character, particularly the mode with the highest intensity in the spectra, which is associated with a symmetric dynamic profile and consequently the largest dynamic stray fields. These findings are supported by both experimental measurements and micromagnetic simulations, offering a consistent and comprehensive picture. Together, the results establish a framework in which the careful design of the geometry and material composition serves as an additional degree of freedom for controlling signal transmission and manipulation at the nanoscale. Furthermore, the studied system reveals a distinct form of magnon–magnon coupling enabled by vertical nanomagnonic design, benefiting the research field of hybrid magnonics.

## Supplementary Material



## Data Availability

Data supporting the findings of this study are available from the corresponding author upon request.

## Notes

Magneto-optical Kerr effect measurements and magnetic parameters for micromagnetic simulations (PDF)

## References

[1] Drisko J., Marsh T., Cumings J. (2017). Topological frustration of artificial
spin ice. Nat. Commun..

[2] Sklenar J., Lao Y., Albrecht A., Watts J. D., Nisoli C., Chern G.-W., Schiffer P. (2019). Field-induced
phase coexistence in an artificial spin
ice. Nat. Phys..

[3] Bhat V. S., Heimbach F., Stasinopoulos I., Grundler D. (2016). Magnetization dynamics
of topological defects and the spin solid in a kagome artificial spin
ice. Phys. Rev. B.

[4] Skjærvo̷ S. H., Marrows C. H., Stamps R. L., Heyderman L. J. (2019). Advances
in artificial spin ice. Nat. Rev. Phys..

[5] Iacocca E., Gliga S., Stamps R. L., Heinonen O. (2016). Reconfigurable wave
band structure of an artificial square ice. Phys. Rev. B.

[6] Gliga S., Iacocca E., Heinonen O. G. (2020). Dynamics of reconfigurable artificial
spin ice: Toward magnonic functional materials. APL Mater..

[7] Lendinez S., Jungfleisch M. B. (2020). Magnetization
dynamics in artificial spin ice. J. Phys.: Condens.
Matter.

[8] Kaffash M. T., Lendinez S., Jungfleisch M. B. (2021). Nanomagnonics
with artificial spin
ice. Phys. Lett. A.

[9] Sultana R. (2025). Ice sculpting: An artificial
spin ice Tutorial on controlling microstate
and geometry for magnonics and neuromorphic computing. J. Appl. Phys..

[10] Zhang W., Xiong Y., Hu J.-M., Sklenar J., Subedi M. M., Jungfleisch M. B., Bhat V. S., Li Y., Liu L., Wang Q., Luo Y. K., Bae Y. J., Flebus B. (2025). Perspective:
Magnon-magnon coupling in hybrid magnonics. arXiv.

[11] Lendinez S., Kaffash M. T., Heinonen O. G., Gliga S., Iacocca E., Jungfleisch M. B. (2023). Nonlinear
multi-magnon scattering in artificial spin
ice. Nat. Commun..

[12] Dion T., Stenning K. D., Vanstone A., Holder H. H., Sultana R., Alatteili G., Martinez V., Kaffash M. T., Kimura T., Oulton R. F., Branford W. R., Kurebayashi H., Iacocca E., Jungfleisch M. B. (2024). Ultrastrong magnon-magnon
coupling and chiral spin-texture control in a dipolar 3D multilayered
artificial spin-vortex ice. Nat. Commun..

[13] Bhat V. S., Jungfleisch M. B. (2025). Magnon signatures of multidimensional
reconfigurations
in multilayer square artificial spin ices. Appl.
Phys. Lett..

[14] Negrello R., Montoncello F., Kaffash M. T., Jungfleisch M. B., Gubbiotti G. (2022). Dynamic coupling
and spin-wave dispersions in a magnetic
hybrid system made of an artificial spin-ice structure and an extended
NiFe underlayer. APL Mater..

[15] Flebus B. (2024). The 2024 magnonics roadmap. J. Phys.: Condens.
Matter.

[16] Montoncello F., Kaffash M. T., Carfagno H., Doty M. F., Gubbiotti G., Jungfleisch M. B. (2023). A Brillouin
light scattering study of the spin-wave
magnetic field dependence in a magnetic hybrid system made of an artificial
spin-ice structure and a film underlayer. J.
Appl. Phys..

[17] Barker O. J., Mohammadi-Motlagh A., Wright A. J., Batty R., Finch H., Vezzoli A., Keatley P. S., O’Brien L. (2024). Thermal nanoconversion
of ferromagnetic nanoislands. Appl. Phys. Lett..

[18] Vogel M. (2015). Optically reconfigurable magnetic materials. Nat. Phys..

[19] Riddiford L. J., Brock J. A., Murawska K., Hrabec A., Heyderman L. J. (2024). Grayscale
control of local magnetic properties with direct-write laser annealing. arXiv.

[20] Gubbiotti, G. Three-Dimensional Magnonics: Layered, Micro- and Nanostructures; Jenny Stanford Publishing: Singapore, 2019. https://www.jennystanford.com/9789814800730/three-dimensional-magnonics/.

[21] Gubbiotti G., Zhou X., Haghshenasfard Z., Cottam M. G., Adeyeye A. O. (2018). Reprogrammable
magnonic band structure of layered Permalloy/Cu/Permalloy nanowires. Phys. Rev. B.

[22] Gubbiotti G., Zhou X., Haghshenasfard Z., Cottam M. G., Adeyeye A. O., Kostylev M. (2019). Interplay between intra-
and inter-nanowires dynamic
dipolar interactions in the spin wave band structure of Py/Cu/Py nanowires. Sci. Rep..

[23] Gubbiotti G., Sadovnikov A., Beginin E., Nikitov S., Wan D., Gupta A., Kundu S., Talmelli G., Carpenter R., Asselberghs I., Radu I. P., Adelmann C., Ciubotaru F. (2021). Magnonic Band
Structure in Vertical Meander-Shaped Co_40_Fe_40_B_20_ Thin Films. Phys. Rev. Appl..

[24] Graczyk P., Krawczyk M., Dhuey S., Yang W.-G., Schmidt H., Gubbiotti G. (2018). Magnonic band
gap and mode hybridization in continuous
permalloy films induced by vertical dynamic coupling with an array
of permalloy ellipses. Phys. Rev. B.

[25] Micaletti P., Roxburgh A., Iacocca E., Marzolla M., Montoncello F. (2025). Magnonic analog
of a metal-to-insulator transition in a multiferroic heterostructure. J. Appl. Phys..

[26] Carlotti G., Gubbiotti G. (2002). Magnetic properties
of layered nanostructures studied
by means of Brillouin light scattering and the surface magneto-optical
Kerr effect. J. Phys.: Condens. Matter.

[27] Sandercock, J. R. Light Scattering in Solids III; Cardona, M. ; Guntherodt, G. , Eds.; Topics in Applied Physics; Springer, 1982.

[28] Vansteenkiste A., Leliaert J., Dvornik, Helsen M., Garcia-Sanchez F., Waeyenberge B. V. (2014). The design and verification of MuMax3. AIP Adv..

[29] Van
de Wiele B., Montoncello F. (2014). A continuous excitation approach
to determine time-dependent dispersion diagrams in 2D magnonic crystals. J. of Physics D: Applied Physics.

[30] Bang W., Montoncello F., Jungfleisch M. B., Hoffmann A., Giovannini L., Ketterson J. B. (2019). Angular-dependent spin dynamics of a triad of permalloy
macrospins. Phys. Rev. B.

[31] Bang W., Montoncello F., Kaffash M. T., Hoffmann A., Ketterson J. B., Jungfleisch M. B. (2019). Ferromagnetic resonance spectra of permalloy nano-ellipses
as building blocks for complex magnonic lattices. J. Appl. Phys..

[32] Goll D., Schutz G., Kronmuller H. (2023). Critical thickness
for high-remanent
single-domain configurations in square ferromagnetic thin platelets. Phys. Rev. B.

[33] Micaletti P., Montoncello F. (2023). Dynamic footprints
of the specific artificial spin
ice microstate on its spin waves. Magnetochemistry.

[34] We extracted the values from the space Fourier transform of the film layer magnon maps of Figure 5b,c, in correspondence of the largest peak of each Fourier spectrum. The wavevector values correspond exactly to wavelength a half and a quarter of the primitive cell size (which is 352 nm).

[35] Venkat G., Kumar D., Franchin M., Dmytriiev O., Mruczkiewicz M., Fangohr H., Barman A., Krawczyk M., Prabhakar A. (2013). Proposal for a Standard Micromagnetic
Problem: Spin
Wave Dispersion in a Magnonic Waveguide. IEEE
Trans. Magn..

[36] Demokritov S. O. (2003). Dynamic
eigen-modes in magnetic stripes and dots. J.
Phys.: Condens. Matter.

[37] Demokritov S. O., Hillebrands B., Slavin A. N. (2001). Brillouin light scattering studies
of confined spin waves: linear and nonlinear confinement. Phys. Rep..

[38] Zivieri R., Montoncello F., Giovannini L., Nizzoli F., Tacchi S., Madami M., Gubbiotti G., Carlotti G., Adeyeye A. O. (2011). Collective
spin modes in chains of dipolarly interacting rectangular magnetic
dots. Phys. Rev. B.

[39] Gubbiotti G., Carlotti G., Okuno T., Grimsditch M., Giovannini L., Montoncello F., Nizzoli F. (2005). Spin dynamics in thin
nanometric elliptical Permalloy dots: A Brillouin light scattering
investigation as a function of dot eccentricity. Phys. Rev. B.

[40] Kittel, C. Introduction to solid state physics, 8th ed.; Wiley: New York, 2005; p 680.

[41] Montoncello F., Tacchi S., Giovannini L., Madami M., Gubbiotti G., Carlotti G., Sirotkin E., Ahmad E., Ogrin F. Y., Kruglyak V. V. (2013). Asymmetry of spin wave dispersions in a hexagonal magnonic
crystal. Appl. Phys. Lett..

