# Supplementary material for: Magnon-Magnon Interaction Induced by Dynamic Coupling in a Hybrid Magnonic Crystal
Source: ACS Appl Electron Mater. 2026 Jan 2;8(1):482–92. doi: 10.1021/acsaelm.5c02128 (PMC12805633; doi:10.1021/acsaelm.5c02128)
Supplement: Supplementary file 1 [file el5c02128_si_001.pdf]

Supporting Information:
Magnon-magnon interaction induced by dynamic coupling in a hybrid magnonic crystal

Rawnak Sultana[1], Mojtaba Taghipour Kaffash[1], Gianluca Gubbiotti[2], Yi Ji[1], M. Benjamin Jungfleisch[1], and Federico Montoncello*[3]

[1]Department of Physics and Astronomy, University of Delaware, Newark, DE19716, USA

[2] CNR-Istituto Officina dei Materiali (IOM), Unità di Perugia, 06123 Perugia, Italy

[3]Dipartimento di Fisica e Scienze della Terra, Università di Ferrara, 44121 Ferrara, Italy

S1. Magneto-Optical Kerr Effect measurements

Magneto-optical Kerr effect (MOKE) magnetometry, which is based on the change in polarization rotation and ellipticity of light upon reflection from a magnetic medium, has proven to be a powerful technique for measuring magnetization curves in magnetic thin films and multilayers.[1] In our experiments, hysteresis loops were measured at room temperature in the longitudinal configuration, i.e., with an applied magnetic field parallel to the sample surface and along the symmetry direction of the ASI. The magnetic field $\mu_0 H$ was swept in the range from -256 to +256 mT in steps of 8 mT. Our MOKE setup consists of a laser source, a photoelastic modulator operating at 50 kHz, and a lock-in amplifier for precise signal detection and noise reduction.

The measured MOKE loops are presented in Fig. S1.

For the unpatterned NiFe film [Sample #1, Fig. S1(a)], the measured MOKE loop exhibits a step-function-like shape, where the magnetization remains nearly saturated over the entire field range, except for a narrow interval (below the adopted field step) around $\mu_0 H$=0, where the magnetization abruptly switches to negative values. The loop for the CoFeB film [Sample #2, Fig. S1(b)] displays a similar shape, but a larger coercivity of approximately 15 mT.

For the isolated CoFeB ASI [Sample #3, Fig. S1(c)], starting from negative saturation ($\mu_0 H$ =−256 mT), the MOKE loop exhibits an elongated shape, characterized by a gradual decrease in magnetization as the field magnitude decreases. This behavior suggests a progressive re-alignment of the magnetic moments in the horizontal islands toward the easy axis (along the x-direction) without

a clear indication of distinct switching fields for the families of CoFeB islands either parallel or perpendicular to the applied magnetic field.

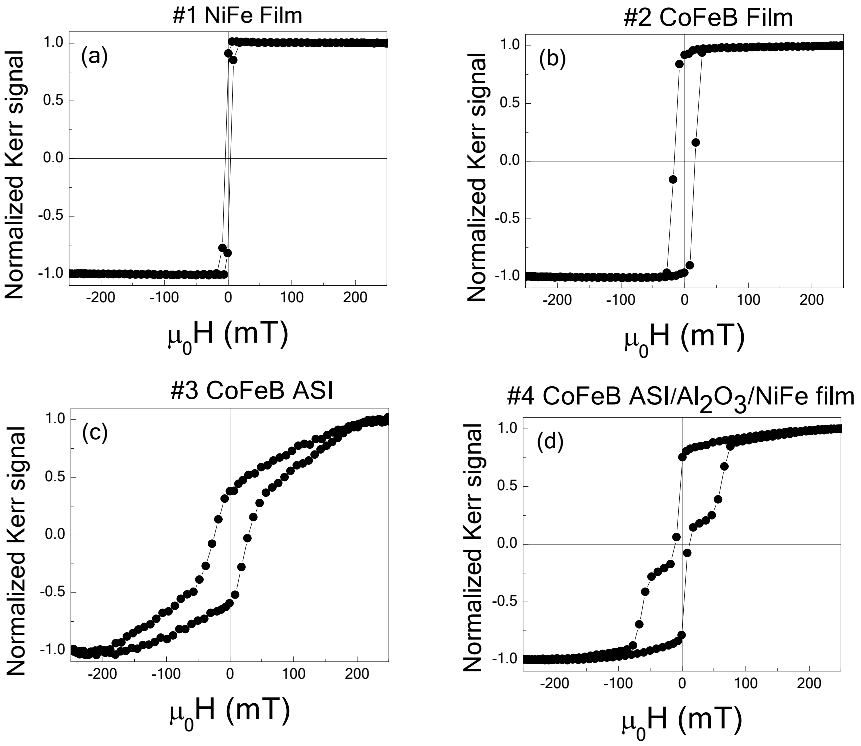

Figure S1. Longitudinal MOKE loops for (a) the continuous NiFe film (Sample #1), (b) the continuous CoFeB film (Sample #2), (c) the ASI array of CoFeB islands (Sample #3) and (d) the hybrid CoFeB ASI/Al_2O_3/NiFe film structure (Sample #4).

For the hybrid system [Sample #4, Fig. S1(d)], starting from $\mu_0 H$=−256 mT, the MOKE loop initially exhibits a gradual increase in magnetization as the field magnitude is ramped up to zero field. This behavior suggests a progressive alignment of the magnetic moments in the vertical islands toward the field direction (x-axis), similar to what is observed for the isolated ASI [Sample #3, Fig. S1(c)]. As the field is swept across $\mu_0 H$=0 mT, a sharp jump in magnetization occurs, which is attributed to the reversal of the continuous NiFe film. For positive fields, the loop features an almost flat plateau, extending from +18 to about 50 mT, followed by a second sharp magnetization switching event associated with the reversal of the vertical CoFeB ASI islands, which are oriented along the direction of the externally applied field. Above 80 mT, a gradual approach to saturation, reached at about 250 mT, is observed. It is noteworthy that, despite the finite penetration depth of light, the MOKE signal is clearly detected from the uncovered regions of the NiFe film that are not overlaid by the ASI

structures. A similar shape of the MOKE hysteresis loop was previously observed in the case of a NiFe ASI patterned on top of a continuous NiFe film.[2]

S2. Magnetic parameters for micromagnetic simulations

The NiFe and CoFeB magnetic parameters, used in the simulations, were obtained by fitting the experimental frequency/wavevector dependence of the unpatterned films with the dipole-exchange surface SW dispersion relation:[3]

$$\omega^2 = \omega_{ex}(\omega_{ex} + \omega_M) + \frac{\omega_M^2}{4}[1 - e^{-2kL}] \quad \text{Eq. (1)}$$

where k is the SW wavevector perpendicular to the applied magnetic field H, i.e., the so called DE[4] configuration, $\omega_{ex} = \gamma\mu_0 H + \lambda_{ex}^2 \omega_M k^2$, with $\omega_M = \gamma\mu_0 M_s$ and $\lambda_{ex}^2 = \frac{2A}{\mu_0 M_s^2}$ (square of the exchange length), and L is the thickness of the continuous reference medium, M_s is the saturation magnetization, A is the exchange stiffness parameter, and $\omega = 2\pi\nu$, where ν is the SW frequency.

From the best fit [Fig. S2(a)], we obtain M_s= 800 kA/m, A =13 pJ/m for NiFe, and M_s=1620 kA/m, A=75 pJ/m for CoFeB. The gyromagnetic ratio was set to γ = 185 rad GHz/T for both materials. We cross-checked the thicknesses of the two thin film layers by treating them as fitting parameters. This resulted in a value of 15 nm, slightly lower than the nominal thickness values measured using a thickness monitor during deposition (likely due to a thin magnetic dead layer).

With the above parameters, the frequency/field curves simultaneously fit the experimental measurements [Fig. S2(b)], showing a monotonic dependence on the applied field with a symmetric behavior for opposite field values, characteristic of the DE SW in single-layer magnetic films. As expected, the measured resonance frequency of the CoFeB film is consistently higher than that of the NiFe film due to the larger saturation magnetization of CoFeB.

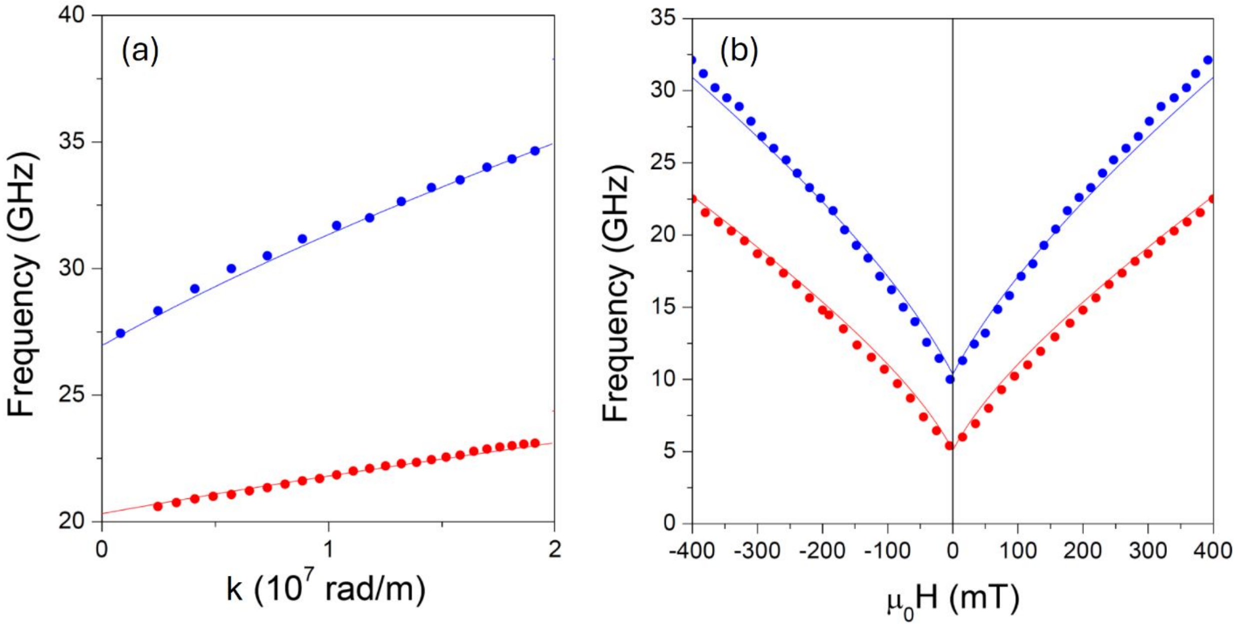

Figure S2. Measured (symbols) and analytical (lines) dispersion curves taken at $\mu_0 H$=350 mT (a), and frequency/field curves taken at an incidence angle of 10° (i.e k=0.41×10^7 rad/m) (b) for the NiFe (red) and CoFeB (blue) reference films. The analytical fits to the experimental data are used to extract the magnetic parameters employed in the micromagnetic simulations.

REFERENCES

[1] Qiu, Z. Q.; Bader, S. D. Surface Magneto-optic Kerr effect (SMOKE). *J. Magn. Magn. Mater.* **1999**, *200*, 664.

[2] Montoncello, F.; Kaffash, M. T.; Carfagno, H.; Doty, M. F.; Gubbiotti; G.; Jungfleisch, M. B. A Brillouin light scattering study of the spin-wave magnetic field dependence in a magnetic hybrid system made of an artificial spin-ice structure and a film underlayer. *J. Appl. Phys.* **2023**, *133*, 083901.

[3] Stancil D. D.; Prabhakar, A. Spin Waves Theory and Applications, 1st ed. New York: Springer-Verlag, 2008.

[4] Damon, R. W.; Eshbach, J. R.; Magnetostatic modes of a ferromangetic slab. J. Phys. Chem. Solids 1961, 19, 308.
